# Human papillomavirus serotypes and determinants among women with invasive cervical cancer in Katsina state, Northwest-Nigeria: a multicentre study

**DOI:** 10.3332/ecancer.2024.1714

**Published:** 2024-06-13

**Authors:** Fatima Abubakar Rasheed, Ibrahim Adamu Yakasai, Aisha Abdurrahman, Asma’u Usman, Nafisat Yusuf

**Affiliations:** 1 Department of Obstetrics and Gynaecology, Federal Teaching Hospital, Katsina 820101, Nigeria; 2 Department of Obstetrics and Gynaecology, Aminu Kano Teaching Hospital, Kano 700233, Nigeria; 3 Department of Anatomic and Molecular Pathology, Federal Teaching Hospital, Katsina 820101, Nigeria; a https://orcid.org/0000-0002-2398-516X; b https://orcid.org/0000-0003-0102-09764; c https://orcid.org/0009-0009-9376-3616; d https://orcid.org/0000-0003-2088-4125; e https://orcid.org/0009-0008-4782-3916

**Keywords:** Human papilloma virus, prevalence, distribution of HPV serotypes, invasive cervical cancer, risk factors

## Abstract

**Background:**

Cervical cancer is the leading cause of gynaecological cancer death among women in developing countries and the most preventable of all gynaecological cancers as its infectious aetiological agent, human papillomavirus (HPV), is known. The knowledge of HPV serotype distribution in a sub-region is key to the implementation of an appropriate HPV vaccination programme.

**Aim:**

To assess the prevalence of HPV-DNA, serotypes and risk-determinants among women with invasive cervical cancer (ICC) in Katsina State, Northwestern Nigeria.

**Methods:**

This was a cross-sectional, multicenter study involving Federal Teaching Hospital Katsina, General Hospital Katsina and Turai Yar’adua Maternal and Child Hospital Katsina, Nigeria. Sixty-three women with histologically confirmed cervical cancer who fulfilled the criteria were recruited into the study. Tissue blocks with a confirmed diagnosis of ICC were taken to DNA Labs Kaduna for HPV-deoxyribonucleotide acid detection and typing. An interviewer-administered questionnaire developed for the study was used to obtain socio-demographic, reproductive characteristics and the other risk factors for HPV acquisition and persistence.

**Results:**

The HPV-positivity rate in ICC was 95.5% while the prevalence of high-risk HPV (Hr-HPV)-DNA in the specimen was 54.6% with 13 HPV-serotypes detected, 9 Hr-HPV types (16,18,31,33,35,45,51,56,82) and 4 low-risk HPV types (6,44,81,89). The most commonly detected HPV serotype among women with a single HPV infection was HPV 81 (40.9%) followed by HPV 16 (28.8%). However, HPV 16 was the most common serotype among those with multiple HPV infections. Prevalence of other detected serotypes were HPV 31 (24.2%), 33 (24.2%), HPV 18 (10.6%), HPV 35 (3.0%), HPV 45 (9.1%), HPV 44 (1.5%), HPV 51 (3.0%), HPV 56 (3.0%), HPV 82 (1.5%), HPV 89 (1.5%) and HPV 6 (1.5%). Forty-four out of 63 women (69.8%) had a single HPV infection, 19 (30.2%) had multiple HPV infections and 15 (24.3%) were co-infected with HPV 16/31/33. There was a statistically significant association between HPV 16 and squamous cell carcinoma (SCC).

**Conclusion:**

The study demonstrates a prevalence of HPV-DNA as 95.5% among women with ICC. The most commonly detected HPV serotype was HPV 81 seen in 41% which was an uncommon finding. Furthermore, statistically significant associations between HPV serotypes 16 and 82 with SCC were detected.

## Introduction

Carcinoma of the cervix is a malignant neoplasm arising from the uterine cervix [1]. Cervical cancer is a significant global health burden and is ranked as the fourth most common cancer among women worldwide and the ninth overall cancer, with an estimated 604,000 new diagnoses and 342,000 deaths that occurred in 2020 [2]. It is the most frequent cancer in 23 countries and the lead cause of cancer mortality in 36 countries most of which is found in sub-Saharan Africa including Nigeria [2]. In Nigeria, cervical cancer is the most common malignancy of the female genital tract with about 14,089 new cancer cases diagnosed yearly and 8,240 women dying from the disease [3]. It accounted for 62.3% [4] of all gynaecological cancers in Kano and 59.3% [5] of all gynaecological cancers in Katsina.

One of the greatest discoveries in human cancer aetiology is the recognition of a causal relationship between human papilloma virus (HPV) and cervical cancer [6, 7]. The precursor lesions usually begin with infection of the metaplastic epithelium of the transformation zone of the cervix with one or more of the high-risk oncogenic human papillomavirus (Hr-HPV) [8]. The invasive spectrum develops only when there is persistent HPV-DNA in the cells [8]. Up to 99.7% of cervical cancers worldwide contain HPV DNA [9, 10].

Approximately 291 million women (10.4%) globally have cervical HPV infection at a point in time [11], which makes timely vaccination with the HPV vaccine an effective primary prevention method. Sub-Saharan Africa has the highest burden of risk factors associated with HPV infection, persistence and progression to cervical cancer.

Prevention and early detection of premalignant states and case findings of more readily treatable cancerous lesions represent critical aspects of a comprehensive strategy to battle cervical cancer effectively but it is limited by adherence to preventive guidelines, suboptimal accuracy of existing screening tests, poor implementation of screening programmes especially in developing countries like Nigeria, as well as wide variance in acceptance rates among our women due to aversion to gynaecological checkups [12, 13].

It is also a known fact that the treatment of gynaecological cancers is one of the greatest challenges we are facing right now in Nigeria [14]. The majority of our hospitals have no established protocol for screening, early diagnosis, functional radiotherapy services or excellent palliative care programme [15]. Our women especially in northern Nigeria have the lowest access to cancer care services due to poverty and late presentation [4, 14, 15]. Screening is still opportunistic and poor in terms of coverage, because of aversion to gynaecological check-ups by our women due to sociocultural and religious [4, 16].

There is a lack of data on HPV prevalence and type distribution due to wide variations in the distribution of these Hr-HPV types throughout the world, which is important to justify the potential benefit of prophylactic vaccines that are currently licensed. In Nigeria, there are only a few large-scale population-based studies on HPV prevalence and subtype distribution reported [17]. There is none on the subtypes from northwestern Nigeria and none from Katsina state and its environs. It was based on the issues highlighted that this study was conceived with the aim to assess the most prevalent HPV serotype among women with invasive cervical cancer (ICC) as well as the risk determinants in Katsina State. Findings from this study will help to make recommendations that may influence the incorporation of routine HPV vaccine into school health programme, as well as, guide the most appropriate vaccination in this region, as part of a strategic framework in eliminating cervical cancer globally by 2030.

## Methodology

The study was a cross-sectional, multicenter study conducted at Federal Teaching Hospital Katsina (FTHKT), General Hospital Katsina and Turai Yar’adua Maternal and Child Health Katsina. The study population comprised aged ≥25 years who had cervical lesions clinically suggestive of ICC attending the gynaecologic clinic, and those referred via gynaecologic emergency unit while, we excluded women who had previously received chemotherapy or radiation for cervical cancer. A sample size of 63 women was obtained using Taylor’s formula for descriptive studies (63 women with invasive cervical carcinoma who have HPV-specific DNA were included in the final analysis).

Eligible women were recruited consecutively using a convenience non-probability sampling technique. Four research assistants were properly trained and handed over the study protocol. The questionnaire was pretested at General Amadi Rimi Special Hospital Katsina using 10% of the estimated sample size.

Written informed consent was obtained from those willing to participate and all eligible women received an interviewer-administered questionnaire to evaluate their socio-demographic characteristics, medical history, gynaecological history as well as HIV status. For those women whose status was unknown, which constituted the majority of the study population, were counselled and offered the test. For standardization, the translated (Hausa version) of the questionnaire was attached so as to standardize the process of data collection.

Examination Under Anaesthesia (EUA), Staging and Biopsy were done by a consultant gynaecologist as part of the routine investigative procedure for any woman with a lesion suspicious of ICC in all three hospitals. The specimens obtained are handled carefully, tagged and appropriately fixed in 10% neutral-buffered formalin (15–20x the volume of the tissue) along with a properly filled histopathology request form, taken to the histopathology laboratory immediately without any delay for processing.

The findings of EUA, the Federation of Gynaecology and Obstetrics cancer stage determined by clinical staging and the histological diagnosis were all recorded on the questionnaire for further analysis.

In the anatomical and molecular pathology laboratory, cervical tissue was grossed to allow the selection of appropriate areas for examination, automated processing of the cervical tissue was done for 24 hours through dehydration in graded strength of alcohol, followed by clearing in xylene, infiltration in molten paraffin wax, embedding in paraffin wax to form tissue blocks and then trimming and sectioning with a microtome (3–5 microns’ thickness). Pathological diagnosis of ICC was determined by light microscopy examination of hematoxylin and eosin-stained sections.

The pathologist ensured all the slides had a second look by another pathologist.

Tissue blocks whose sections were diagnosed as ICC were moved to the DNA labs in Kaduna where HPV-DNA detection and typing were done. Deoxyribonucleotide acid was extracted from Formalin-Fixed Paraffin-Embedded (FFPE) tissues using Accuprep Genomic DNA Extraction Kit from Bioneer (Daejeon, South Korea) according to the manufacturer’s instruction [18].

The eluted genomic DNA was run for human β-globin as a house keeping gene to ascertain the quality and viability for polymerase chain reaction (PCR) detection of HPV using (Accupower Hotstart PCR premix, Bioneer). A primer that targets a 122-bp sequence of the β- globin was used; Forward: 5′CTTCTGACACAACTGTGTTCACTAGC3′, Reverse: 5′TCACCACAACTTCATCCACGTTCACC 3′. This study utilized AccuTarget Real-Time PCR Kit from Bioneer corporation. The β-globin-positive DNA samples were subjected to PCR to detect the HPV-DNA using SPF10 consensus primers. Amplification was detected using agarose gel electrophoresis. A third round was allowed to run using the 2nd round primers in those samples whose amplicon is not visible.

From this stage, HPV-positive specimens were typed by reverse hybridization line probe assay using 27 type-specific hybridization probes (LiPA25), which detect 14 high-risk (HPV16, HPV18, HPV31, HPV33, HPV35, HPV39, HPV45, HPV51, HPV52, HPV56, HPV58, HPV59, HPV66, HPV68 = 73) and 11 low-risk (HPV6, HPV11, HPV34, HPV40, HPV42, HPV43, HPV44, HPV53, HPV54, HPV70, HPV74, HPV 81, HPV 82) types, as previously described. The DNA of HPV-negative samples was diluted up to ten times and then re-tested to rule out false-negative results. HPV-DNA genotyping was done using sequencing. Sequences of each sample were typed into the blasting software and the HPV type result was obtained.

Data obtained were entered into Microsoft Excel for cleaning and analysed using Statistical Package for Social Sciences version 28. Qualitative variables were summarised as frequencies and percentages while quantitative variables were summarised as means and standard deviations. Bivariate analysis was used to test for association between variables (Chi-square or Fishers exact test). The level of statistical significance was set at *p* < 5% (0.05).

Ethical approval was obtained from the ethics committees of FTHKT and the State Ministry of Health (FTHKTHNREC no: N003/082012; Katsina HREC no: MOH/ADM/SUB/1152/1/483). The provisions of the Helsinki Declaration were adhered to throughout the study.

## Results

**Figure 1.**
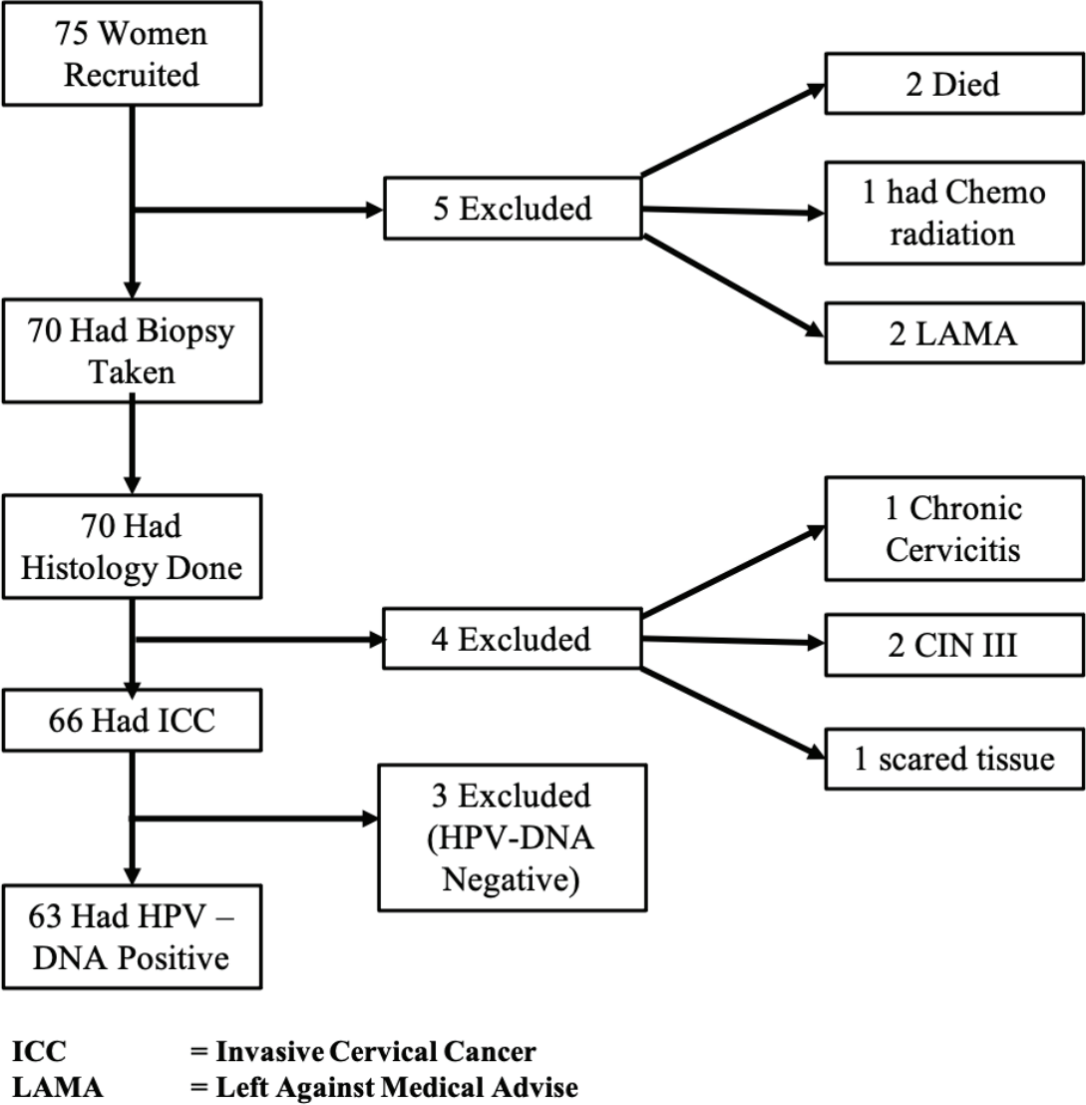
Patient flow chart from recruitment to final stage of analysis.

Figure 1 depicts the client flow chat for this study. A total of 75 women who had cervical lesions clinically suggestive of ICC attending the gynaecologic clinic, colposcopy clinic and those referred via gynaecologic emergency unit were assessed. Two women left against medical advice before EUA/Staging/Biopsy was done for them; one gave a prior history of chemo-radiation at Department of Radiotherapy and Oncology, Ahmadu Bello University Teaching Hospital; two died while on admission before EUA/Staging/Biopsy was done and four histology were negative for ICC (2 were CIN III,1 was a scar from previous cervical injury from an unsupervised home deliver and 1 was chronic cervicitis). Sixty-six women had histologically confirmed ICC (Study Population) and were tested for HPV-DNA. Out of the 66 women, 63 women with invasive cervical carcinoma were found to have HPV-specific DNA. Three blocks (10, 34 and 40) were negative for HPV-DNA.

Table 1 summarizes the socio-demographic characteristics of the women in this study. The mean age of the women in the study was 53.3 ± 13.48 years, with a range of 26–80 years. The majority (25.8%) were within the age range of 45–54 years. Ninety-Eight-point five percent (65) of the study population were Hausa/Fulani by tribe, only 1 was Yoruba with none from the other tribal groups. A great majority (98.5%) belonged to the Islamic faith. Fifty-nine point one percent reported no form of education, and only seven women (10.6%) had education up to tertiary level. Most (81.8%) were unemployed and about 10% were civil servants. Women of low social class constituted the majority of the study population.

Table 2 summarizes the sexual and reproductive characteristics of the study population. Most (92.1%) were HIV negative. Though the majority (77.27) were married, a significant number (22.73) were divorced/widowed. Among the married ones, 18.2% were in their 3rd order of marriage and above. 90.9% have no knowledge of Pap smear in the past, still 90.9% were never screened in the past. Most (95.5%) neither used any form of contraception nor used injectable and only 3 women (4.5%) have ever used COCP in their lifetime. Eighty-three-point-three percent were grand multiparous women. The mean age at coitarche was 15.06 ± 1.77 with a range of 12–26 years. Fifty-one (77.3%) had only one sexual partner.

Table 3 summarizes the distribution of HPV among women with ICC who were HPV positive. HPV 81 was seen among 27 (40.9%) of the HPV-positive women. HPV 16, 31 and 33 were equally commonly seen among 19 (28.8%) of the women for HPV 16 and then 6 (24.2%) women each for HPV 31 and 33. HPV 18 was seen exclusively among 7 (10.6%) women. Sixteen women had HPV 16,31,33 co-infection together.

**Figure 2.**
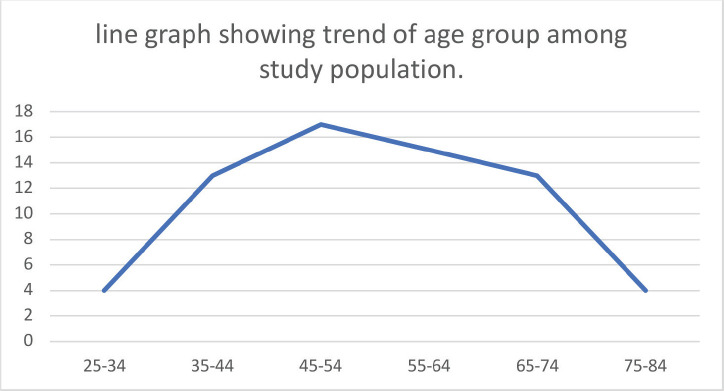
Line graph showing trend of age group among study population.

Table 4 summarizes the distribution of HPV types (*n* (%)) among women with histologically confirmed ICC who were infected with single and multiple HPV types as depicted in Figure 2 above. Forty-four (69.8%) had a single infection while 19 (30.2%) had multiple infections. The most frequently detected HPV types among women with multiple infections was HPV16, 31 and 33. Of the 44 women who had a single infection, HPV 81 accounted for 52.3% of cases of single HPV infection. Co-infection with HPV 16/31/33 occurred 16 times (84.2%).

Table 5 summarizes the histologically confirmed tumour type among the women. Of the 66 women, squamous cell carcinoma (SCC) was the most commonly diagnosed seen among 77.3% (51). The keratinizing form was the most common and accounted for 78.4% of the SCC type and 60.6% of whole histological types. There were five cases of ASC, two cases of ADC, four had microinvasive squamous carcinoma. Of the four others; one was histologically confirmed transitional cell carcinoma in a 33-year-old woman, 1UDC, I was PDC.

Table 6 shows the association between some socio-demographic, reproductive and sexual characteristics between those women with histologically confirmed ICC who were positive for HPV-DNA and those who were negative for HPV-DNA. There was no statistically significant difference in the mean age of the women across the two groups. The mean ages were 53.5 ± 13.49 for women positive for HPV-DNA and 48.3 ± 14.84 for those negative for HPV-DNA. The mean age at coitarche was similar across the two groups (15.1 ± 1.81 versus 15.3 ± 0.58). Other variables in the table (Educational status, Marital order, Knowledge of Pap smear, usage of COCP, History of smoking, their HIV Status and social class) were not significantly different between the two groups.

Table 7 summarizes the distribution of HPV types according to tumour type in women with histologically confirmed ICC infected with a single HPV infection. There was a statistically significant association between the presence of HPV 16 in the tumour of those with histologically confirmed SCC, *p* < 0.05*.* HPV 82 was statistically associated with histological diagnosis of transitional cell carcinoma, *p* < 0.05.

## Discussion

This cross-sectional study used Nested PCR with a different set of primers for the first round (PGMY 11 primer set), second round (GP5&6) and unique to this study, was a third round using second set primers to detect the presence of HPV type-specific DNA to assess the prevalence, type distribution and associated factors of HPV infection among women with histologically confirmed ICC in Katsina state, Nigeria.

The socio-demographic characteristics of women in this study show a maternal age preponderance of 50–59 years with a mean age of 53.27, similar to the global finding of an average age at diagnosis of ICC of 53 years [19]. This was comparable to the finding of, 51 years from Shika-Zaria, [20] Kano, [21] Sokoto, [16] and from the works of Denny *et al* [22] (58 years in Nigeria, 56 years for Ghana and 51 years for South Africa). The majority (98.5%) of the study population were Hausa/Fulani by tribe, and were of Islamic faith, 59.1% had no formal education and were unemployed. These findings were similar to findings in Northern-Nigerian sub-region from several studies [4, 16, 19, 20, 23].

The prevalence of HPV- DNA positivity was 95.5% in this study. This is not surprising as the study population were women with histologically confirmed ICC, literatures have also reported that, the invasive spectrum of the disease develops only when there is persistent HPV-DNA in the cells [6, 8] . For this study, freshly made FFPE tissues blocks were shifted to the molecular laboratory for DNA extraction and subsequent typing, this could have contributed to a higher DNA extraction. Another plausible explanation for a high prevalence of 95.5% was those samples at DNA Labs that showed no or faint band at the molecular laboratory were subjected to a third round of PCR using second set primers (GP5+/6 +-mediated PCR) until a visible band was seen (33 out of the 36 samples gave a clear and dense visible band after the third round). Studies using PGMY instead of MY primers have the potential to report higher detection rates of HPV infection not only for well-known genital types but for newer HPV types but also, this index study employed the use of the novel PGMY L1 consensus primer pair.

The findings in the index study are similar to the 90.4% from the works of Denny *et al* [22] using a similar methodology. It was also comparable to a prevalence of 85.6% [17] from Lagos, 76.92% [24] from Benin republic, 89.8% [25] from Ghana and 92.5% [26] from Morocco, 95.8% [27] from UK. However, a slightly lower prevalence of 69.8% was reported by Kabir *et al* [28] with a similar methodology to the index study but with the utilization of archival blocks which could be the plausible reason for their lower rate as a significant degradation of their DNA may have occurred in archival blocks after 4–6 years of storage [29]. The prevalence of the index study is comparable to 93% [30] among cases of ICC from India and 97.6% [31] in SCC of women with ICC from China.

In contrast to our finding and most findings reported in Africa, some studies, however, reported a low prevalence of HPV among ICC notably was Egypt which reported 22.6%, and another study 2 years later 33.3% [32, 33]. When they compared with prevalence in pre-malignant lesions, the prevalence was comparable, they concluded that, they are yet to determine the usefulness of HPV vaccination in their area [32].

In the index study, the most frequently detected HPV serotype among women with histologically confirmed ICC was HPV 81 with a prevalence of 40.9%. HPV 16 was the second most common among women infected with a single HPV-type infection with a prevalence of 28.8%. While HPV 16 dominated as the most common multiple infection consistently occurring with 31 and 33 in 84.2% of the cases. This finding of a high prevalence of HPV 81 was least expected but an important finding in this study as HPV 16 and /or 18 were the serotypes reported as the most frequently detected in most Sub-Saharan African studies with relative contributions ranging from 56.4% to 91.5% [18]. This is the first-ever study in the Northwestern Nigeria. Anorlu and colleagues in 2014 from Lagos with a similar methodology found HPV 16 as the commonest among women with positive histological diagnosis of cervical cancer. Equally, similar studies conducted in south-west Nigeria, [17] and in Maiduguri (north-east), [27] also reported HPV 16 as the commonest HPV detected. Interestingly, non-16/18 Hr-HPV type were found to be higher among black women of African descent presented at the Canadian Academy of Pathology 2020 Annual Meeting [34].

However, in this study, HPV 16 was the second most common, and HPV 18 ranked 5th. It is also in contrast to what was found in places such as Irwa [35] and Abuja [36], where HPV 35 is the most common in Ghana HPV 59 was the most common HPV seen in cervical cancer, [37] while HPV-16 as most prevalent in Kenya [38].

No study reported the finding of HPV 81 within Nigeria in ICC. HPV 81 is now recently among the 3 HPV previously classified by the International Agency for Research on Cancer as intermediate risk-HPV that were now re-classified as ‘potentially high-risk’ HPV (PHR-HPV) because of their isolation in several samples of SCC in women and based on their phylogenetic similarity to other high-risk type [39–41]. The emergence of various commercial HPV genotyping kits with different characteristics facilitates the detection of most high-risk and low-risk HPV genotypes, but the rare HPV types are usually underdiagnosed like HPV 81 [37]. In the present study, HPV detection was performed using *PCR-REAL TIME(RT) (Accupower Hotstart PCR premix, Bioneer)* which using several primers that detected 44 serotypes including both Hr-HPV and Lr-HPV*.* Results of some Emerging studies suggested the potential roles of some rare HPV types, one of which is HPV 81 in cervical lesions and cervical cancer [38]. Several other HPV types (26, 30, 34, 53, 66, 67, 69, 70, 73, 82, 85, and 97) are possibly carcinogenic based on evolutionary similarity to the known cancer-causing types [38, 40]. HPV 81 type is now being detected frequently in samples with CIN III [39]. Among the Han Ethnic group, they reported finding a high prevalence PHR-HPV 81 in SCC lesions among their women. However, a lower prevalence of 29% HPV 81 was reported in cervical lesions living in Ecuador and 4.6% in the eastern region of Uttar Pradesh India [42].

The contribution of HPV 45 to ICC in this study is similar to the finding that, ‘two additional serotypes were frequently detected in this sub-Saharan African study: HPV45 and HPV35’ [27]. HPV45 is being identified as a common type in a global, retrospective, cross-sectional study conducted among 544 women in Africa with histologically confirmed ICC and other studies [21]. Also, the relative high contribution of HPV 31 and HPV 33 with their occurrence with HPV 16 in all instances as co-infection in women with multiple HPV infection could be a phenomenon unique to this area of the country which is an area to look into. Surprisingly, only 1 patient had HPV 16/18 together in this study in contrast to the findings of Denny *et al* [21].

The risk factors for ICC have been well established and include, HPV which is the most important risk factor in disease progression to uterine cancer as 90% and more of cervical carcinomas retain HPV DNA in the malignant cells phenotype [6, 43, 44]. The second category are the demographic, personal or sexual risk factors which include early age at coitarche, multiple lifetime sexual partners, high parity, history of sexually transmitted diseases, smoking, chronic immunosuppression, history of genital warts, never or infrequent Pap testing and prolonged use of oral contraceptives [45]. All these factors were assessed in this present study and HPV DNA was found to be retained in 95.5% of the malignant cell.

A relatively high percentage-83.3% of women in this study were grand multiparous. Multiparity raises the (RR = 3.8) [44]. Although, literature has reported a dose-dependent increase in risk with numbers of live births. This epidemiologic association is firmly established, the explanatory mechanism is not quite clear.

In this study, 19.7% have smoked cigarette, mean estimate of pack-year was 10.25 pack-year among the women with ICC in this study. Up to 19.7% have ever smocked tobacco with 66% smoking ten pack-year or more in their life time among the women with ICC in this index study. This finding of a significant number of cigarette smokers among cervical cancer is similar to finding in several studies that reported tobacco smoking as an independent risk factor for HPV-DNA persistent and ICC [46–48].

Use of COCP for more than 5 years (RR = 1.90) [49, 50]and for more than 10 years (OR = 4.48; 2.24 to 9.36) [7]. The conclusion of this analysis indicates that the use of oral contraceptives for 5 or more years is a cofactor that increases up to fourfold the risk of cervical cancer among women who are carriers of HPV DNA. In this present study, only three used COCP, none has ever used it continuously for 5 years among the study population.

HIV infection is strongly associated with HPV infection. Several studies have shown that higher prevalence and persistence of HPV are all more frequent in HIV positive women [21, 46, 51]. In this study, only 5 (7.5) were HIV-Positive. 92.4% were HIV negative. This relatively low HIV prevalence even among women with ICC is not surprising as a recently published Nigeria HIV/AIDS indicator and impact survey, one of the largest population-based HIV/AIDS house hold surveys ever conducted, found a prevalence of HIV as just 1.4% with katsina having the lowest prevalence; 0.6% [52, 53].

In this study, 68.3% are of low social class. Several international meta-analyses have published that, women defined as belonging to a low socio-economic (reflecting poverty, poor educational status (79.3% had no formal education) class were found to have twice the risk of ICC compared to those in a high socio-economic class, and a lower than average participation in Pap smear screening [4, 54, 55]. Although there was no statistically significant difference in terms of social class between the two group in our study, it showed a strong association between low socio-economic status and ICC.

Even before data convincingly linked HPV to the development of cervical cancer, it was well recognized that various aspect of a patient’s sexual history put women at increased risk for developing pre-invasive and subsequent invasive lesions of the cervix [7]. Early age at first intercourse (AFSI) which was regarded as having had first sexual intercourse at or before age 14 years was 92.4% among the study population, AFSI (RR is twofold for younger than 18 years compared to 21 years old. The finding of AFSI in this study is similar to findings reported in many studies in Nigeria, and Sub-Saharan African sub region [17, 21, 56].

Another important finding in this study was the higher prevalence (77.3%) of single life-time sexual partners among women with histologically confirmed ICC compared to 22.7% who had two or more life-time sexual partners. The literature has reported that multiple sexual partners is a risk factor for developing cervical cancer [1, 48]. Several studies also found increasing number of sexual partners as a co-factor for ICC, [57, 25, 44]which is in contrast to what we found in this index study. In this study, the role of male sexual behaviour was not assessed. Male sexual partners play a role in cervical carcinogenesis [58]. Also, in an observation made over a century ago and a scientific hypothesis formulated almost 30 years ago that male sexual behaviour is a central determinant of the incidence of cervical cancer in populations where female monogamy is dominant such as ours [55]. As such male behaviour in cervical carcinogenesis among women in this sub-region who had one lifetime sexual partner may be an important aspect to look into. This important finding may pave the way for further research in this region which might change the narrative and notion that multiple partners are more important factor than single partner or discard it. However, there may be under-reporting of the number of life time sexual partners, especially in areas like katsina where having multiple sexual partners is frowned upon for women. The high figure of 77.3% may be actually less than it is. this could be a limitation of the study as truly the number of life time sexual partners may not be a true reflection of the reality. It is worthy to note that, while multiple sexual partners are a known risk factor for cervical cancer, a history of a single sexual partner does not exclude the risk of HPV and cervical cancer.

In this index study also, HPV16 predominated in women with SCC similar to findings from in keeping with other recent studies on the distribution of HPV in SCC in Nigeria, Ghana, India, UK [21, 24, 29, 26]. There was also a statistically significant association with HPV 82 and SCC. In terms of ADC and ASC, there was no statistically significant difference in terms of predominance of HPV-serotype. This contradicts the findings from Nigeria, Ghana and south Africa and other recent studies which reported than HPV 18, HPV 45 were frequently identified in samples of ADC [21]. The finding from this study did not show a statistically significant association between HPV 81, the most frequently detected HPV serotype with any histological type of ICC.

There was one case each for transitional cell carcinoma and undifferentiated SCC and all had a single HPV infection with 81.

## Conclusion

This study, the first to be reported in this region; shows a significantly high prevalence of HPV-DNA among women with histologically confirmed ICC with a value of 95.5%. HPV 81 was the most frequently detected serotype seen among 27 (40.9%) of the women who were positive for HPV-DNA, followed by HPV 16 (28.8%), HPV 31 and HPV 33 (24.2% each), then HPV 18 (10.6%). There was no statistically significant difference among women with histologically confirmed ICC that were positive for HPV-DNA and the group that were negative for HPV-DNA in terms of socio-demographic, sexual and reproductive characteristics, but the histomorphological pattern was found to have a statistically significant association with HPV-serotype (there was a statistically significant association between HPV 16 and 82 serotypes with SCC).

- It will therefore be beneficial to conduct a bigger multicenter study with a larger sample size across the country to establish the serotypes in each geopolitical zone and study the disease progression of HPV 81 in both pre-invasive and ICC, especially in Northwestern Nigeria.
- The feasibility of developing a polyvalent vaccine incorporating this additional HPV 81 from the result of a larger study for our sub-region.
- With the poor knowledge of cervical cancer screening and its uptake, Sensitization programmes on cervical cancer screening, the role of vaccination as a primary preventive measure especially targeting the adolescent group should be at the forefront.

## List of abbreviations

FTHK, Federal Teaching Hospital Katsina; GLOBOCAN, Global Cancer Estimates; HREC, Health Research Ethics Committee.

## Conflicts of interest

There is no conflict of interest.

## Funding

There was no funding or sponsorship for this study.

## Author contributions

The authors have met the criteria for authorship according to the ICMJE. Rasheed FA and Yakasai IA conceptualised and designed the study. The proposal was written by Rasheed FA and reviewed by Yakasai IA. Patient recruitment, sample preparations, data collection, were performed by Rasheed FA and Yusuf NA. The laboratory aspect of the work was handled by Usman A. Data analysis was performed by Rasheed FA and Abdurrahman A. Results were written by Rasheed FA and Usman A. Discussion was written by Rasheed FA and Abdurrahman A. The first draft of the manuscript was written by Rasheed FA and critically reviewed by Yakasai IA for important intellectual content. The authors commented on previous versions of the manuscript. The authors have read and approved the final manuscript for publication. The authors who do not met the criteria for authorship have been duly acknowledged.

## Figures and Tables

**Table 1.** Sociodemographic characteristics of study population (*N* = 66).

Variables	Frequency (*n* = 66)	Percentage (%)
Maternal age (years)		
20–29	1	1.5
30–39	13	19.7
40–49	10	15.2
50–59	18	27.3
60–69	12	18.2
70–79	10	15.2
80–89	2	3.0
Total	66	100
Mean age ± SD	53.27 ± 13.48	
Range	26–80	
Ethnic group		
Hausa	65	98.5
Yoruba	1	1.5
Total	66	100
Religion		
Islam	65	98.5
Christianity	1	1.5
Total	66	100
Educational status		
None	39	59.1
Quranic	13	19.7
Primary	3	4.5
Secondary	4	6.1
Tertiary	7	10.6
Total	66	100
Occupation		
Unemployed	54	81.8
Self-employed	6	9.1
Civil servants	6	9.1
Total	66	100
Social class		
High	7	10.6
Middle	16	24.2
Low	43	65.2
Total	66	100

**Table 2.** Sexual and reproductive characteristics of women with ICC.

Variables	Frequency (*n* = 66)	Percentage (%)
HIV status		
Positive	5	7.6
Negative	61	92.4
Total	66	100
Smoking		
Yes	13	19.7
No	53	80.3
Total	66	100
Marital status		
Married	51	77.27
Single	0	0.0
Divorced/widowed	15	22.73
Total	66	100
Marital order		
1st	30	45.5
2nd	24	36.4
≥3rd.	12	18.2
Total	66	100
Knowledge of Pap smear		
Yes	6	9.1
No	60	90.9
Total	66	100
Previous screening		
Yes	6	9.1
No	60	90.9
Total	66	100
Previous COCP usage		
Yes	3	4.5
No	63	95.5
Total	66	100
Parity		
1	0	0.0
2–4	11	16.7
≥5	55	83.3
Total	66	100
Age @ Coitarche		
≤16	61	92.4
>16	5	7.6
Total	66	100
^a^Early age at 1st intercourse (AFSI) = 92.4%		
		
Range	12–26	
Mean ± SD	15.06 ± 1.77	
		
Number of life time sexual partners		
1	51	77.3
≥2	15	22.7
Total	66	100

a percentage of women that commenced sexual debut at or before the age of 14 years

**Table 3.** Distribution of HPV type-specific among HPV-positive women with ICC.

HPV serotype	Frequency (*n* = 66)	Percentage (%)
6	1	1.5
16	19	28.8
18	7	10.6
31	16	24.2
33	16	24.2
35	2	3.0
44	1	1.5
45	6	9.1
51	2	3.0
56	2	3.0
81	27	40.9
82	1	1.5
89	1	1.5

**Table 4.** Single and multiple HPV infections among HPV-positive women with ICC.

Serotypes	Single infection (*n* = 44(%))	Multiple infection (*n* = 19(%))
HPV 6	0 (0.0)	1 (5.3)
HPV 16	3 (6.8)	16 (84.2)
HPV 18	6 (13.6)	1 (5.3)
HPV 31	0 (0.0)	16 (84.2)
HPV 33	0 (0.0)	16 (84.2)
HPV 35	1 (2.3)	1 (5.3)
HPV 44	1 (2.3)	0 (0.0)
HPV 45	6 (13.6)	0 (0.0)
HPV 51	1 (2.3)	1 (5.3)
HPV 56	2 (4.6)	0 (0.0)
HPV 81	23 (52.3)	3 (15.8)
HPV 82	1 (2.3)	0 (0.0)
HPV 89	0 (0.0)	1 (5.3)

**Table 5.** Histological types distribution among women with ICC.

Histology	Frequency (*n* = 66)	Percentage (%)
LCK SCC	40	60.6
LCNK SCC	11	16.7
ASC	5	7.46
ADC	2	3.28
MIC	4	6.06
Others	4	6.06
Total	66	100

Key: Squamous cell carcinoma (SCC) (LCK= Large cell keratinizing, LCNK= Large cell non keratinizing). Microinvasive Squamous Cell Carcinoma (MIC), adenosquamous carcinoma (ASC), adenocarcinoma (ADC). Others (transitional cell carcinoma, undifferentiated squamous cell carcinoma and poorly differentiated squamous cell carcinoma)

**Table 6.** Relationship between HPV-DNA type specific and possible associated factors among women with ICC.

Variable	Positive for HPV-DNA (*n* = 63), *n* = (%).	Negative for HPV-DNA (*n* = 3), *n* = (%).	Test statistics	*p*-value
Mean age ± SD (years)	53.5 (13.49)	48.3 (14.84)	*t* = 0.647	0.520
Mean age @ Coitarche ± SD (years)	15.1 (1.81)	15.3 (0.58)	*t* = -0.271	0.787
Educational status				
None	38 (60.3)	1 (33.3)	χ^2^ = 2.514	0.642
Quranic	12 (19.0)	1 (33.3)		
Primary	3 (4.8)	0 (0.0)		
Secondary	4 (6.3)	0 (0.0)		
Tertiary	6 (9.5)	1 (33.3)		
Marital order				
1st	28 (44.4)	2 (66.7)	χ^2^ = 0.890	0.641
2nd	23 (36.5)	1 (33.3)		
≥3rd	12 (19.0)	0		
Knowledge of Pap smear				
Yes	6 (9.5)	0 (0.0)	Fisher’s exact	1.000
Never	57 (90.5)	3 (100.0)		
COCP usage				
Yes	3 (4.8)	0 (0.0)	Fisher’s exact	1.000
No	60 (95.2)	3 (100.0)		
History of smoking				
Yes	13(20.6)	0 (0.0)	Fisher’s exact	1.000
No	50(79.4)	3 (100.0)		
HIV status				
Yes	5 (7.9)	0 (0.0)	Fisher’s exact	1.000
No	58 (92.1)	3 (100.0)		
Parity				
Multigravida	9 (14.3)	2(66.7)	Fisher’s exact	0.070
Grand multiparous	54(85.7)	1(33.3)		
Social class				
High	6 (9.5)	1 (33.3)	χ^2^ = 5.912	0.052
Middle	14 (22.2)	2 (66.7)		
Low	43 (68.3)	0 (0.0)		

*p < 0.05* are statistically significant, COCP = Combined oral contraceptive pills, SD = Standard deviation.

χ^2^ = CHI-SQUARE. *t* = student *t*-test

**Table 7.** Distribution of HPV types according to tumour type in women with histologically confirmed ICC infected with a single HPV type.

HPV serotypes	SCC (*n* = 51(%))	ASC/ADC (*n* = 7(%))	Others (*n* = 8(%))	Test statistics	*p*-value
HPV 6					
Positive	1 (2.0)	0 (0)	0 (0)	χ^2^ = 0.299	0.861
Negative	50 (98.0)	7 (100.0)	8 (100.0)		
HPV16					
Positive	38 (74.5)	3 (42.9)	3 (37.5)	χ^2^ = 7.362	0.025
Negative	13 (25.5)	4 (57.1)	5 (62.5)		
HPV18					
Positive	6 (11.8)	0 (0)	1 (12.5)	χ^2^ = 0.933	0.627
Negative	45 (88.2)	7 (100)	7 (87.5)		
HPV31					
Positive	12 (23.5)	3 (42.9)	2 (25.0)	χ^2^ = 1.205	0.547
Negative	39 (76.5)	4 (57.1)	6 (75.0)		
HPV33					
Positive	12 (23.5)	3 (42.9)	2 (25.0)	χ^2^ = 1.205	0.547
Negative	39 (23.5)	4 (52.9)	6 (75.0)		
HPV35					
Positive	2 (3.9)	0 (0)	0 (0)	χ^2^ = 0.607	0.738
Negative	49 (96.1)	7 (100.0)	8 (100.0)		
HPV44					
Positive	1 (2.0)	0 (0)	0 (0)	χ^2^ = 0.299	0.861
Negative	50 (98.0)	7 (100.0)	8 (100.0)		
HPV45					
Positive	4 (7.8)	1 (14.3)	1 (12.5)	χ^2^ = 0.437	0.804
Negative	47 (92.2)	6 (85.7)	7 (87.5)		
HPV51					
Positive	2 (3.9)	0 (0)	0 (0)	χ^2^ = 0.607	0.738
Negative	49 (96.1)	7 (100.0)	8(100.0)		
HPV56					
Positive	2 (3.8)	0 (0)	(0)	χ^2^ = 0.607	0.738
Negative	49 (96.1)	7 (100.0)	8 (100.0)		
HPV81					
Positive	23 (45.1)	3 (42.9)	1 (12.5)	χ^2^ = 3.052	0.217
Negative	28 (54.9)	4 (57.1)	7 (87.5)		
HPV82					
Positive	0 (0)	0 (0)	1 (12.5)	χ^2^ = 7.996	0.015
Negative	51 (100.0)	7 (100.0)	7 (87.5)		
HPV89					
Positive	1 (2.0)	0 (0)	0 (0)	χ^2^ = 0.299	0.861
Negative	50 (98.0)	7 (100.0)	8 (100.0)		

*p* < 0.05 are statistically significant, *χ*^2^ = Chi-square test

Key: squamous cell carcinoma (SCC (LSK SCC & LSNK SCC)). Adenosquamous carcinoma (ASC), Others (transitional cell carcinoma, undifferentiated squamous cell carcinoma and poorly differentiated squamous cell carcinoma.)

**Slide 1.**
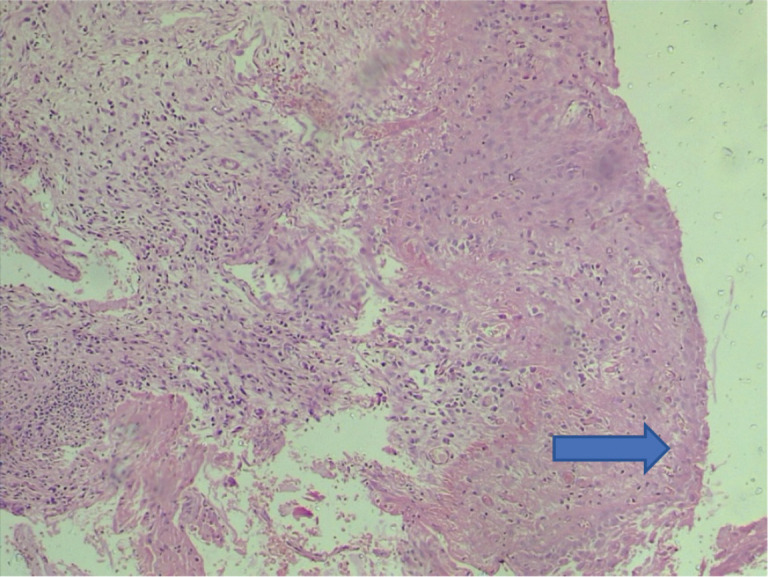
Photomicrograph of a micro invasive squamous cell carcinoma of the cervix showing the tumour cells breaching the basement membrane (arrow). H & E. Magnification X100. From block 5. (Photomicrographs taken from slides of some of the women in this study).

**Slide 2.**
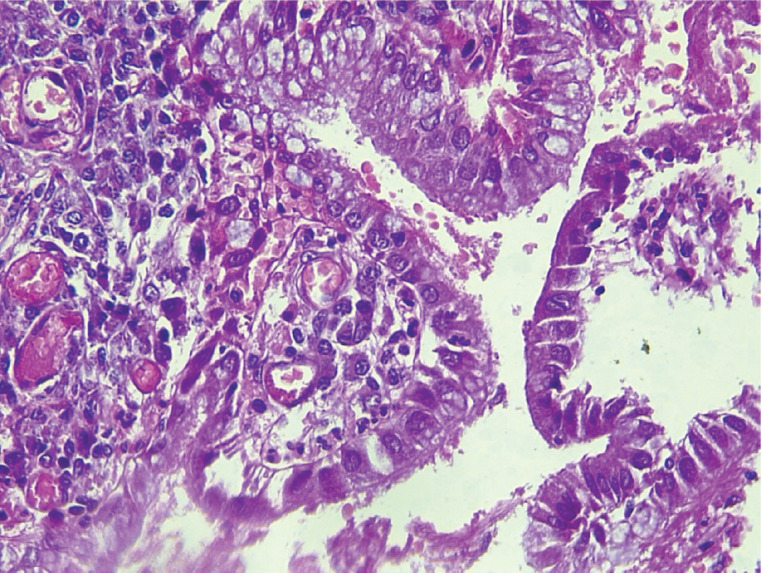
Photomicrograph of an adenocarcinoma of the cervix showing atypical endocervical glands exhibiting hyperchromasia and loss of polarity. H & E. Magnification X400. From block 2. (Photomicrographs taken from slides of some of the women in this study).

**Slide 3.**
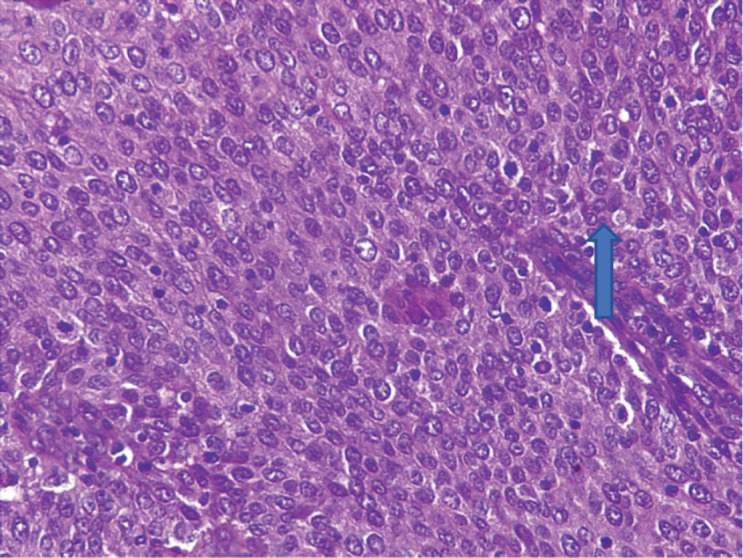
Photomicrograph of a large cell non-keratinizing squamous cell carcinoma of the cervix showing moderately pleomorphic polygonal cells and frequent mitoses (arrow). H & E. Magnification X400. From block 51. (Photomicrographs taken from slides of some of the women in this study).

**Slide 4.**
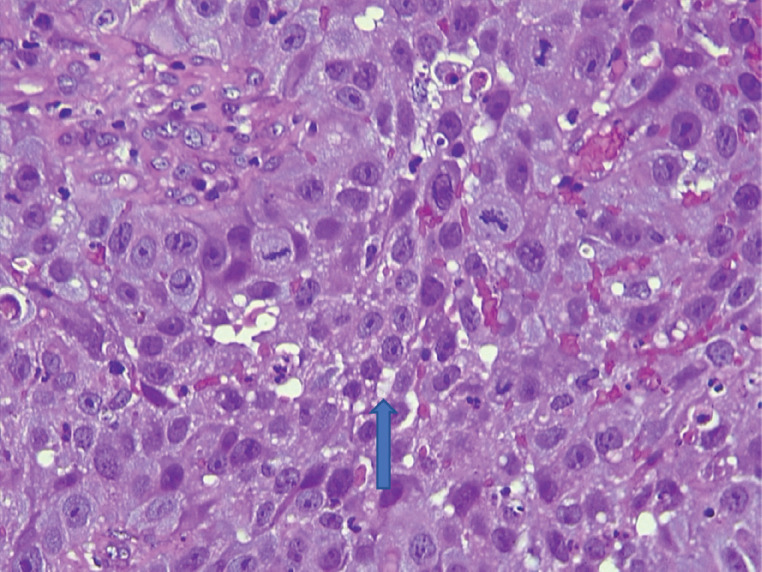
Photomicrograph of a large cell keratinizing SCC of the cervix showing atypical squamous cells with individual cell dyskeratosis (arrow) and brisk mitotic activity. H & E. Magnification X400. From block 56. (Photomicrographs taken from slides of some of the women in this study).

**Figure A1.**
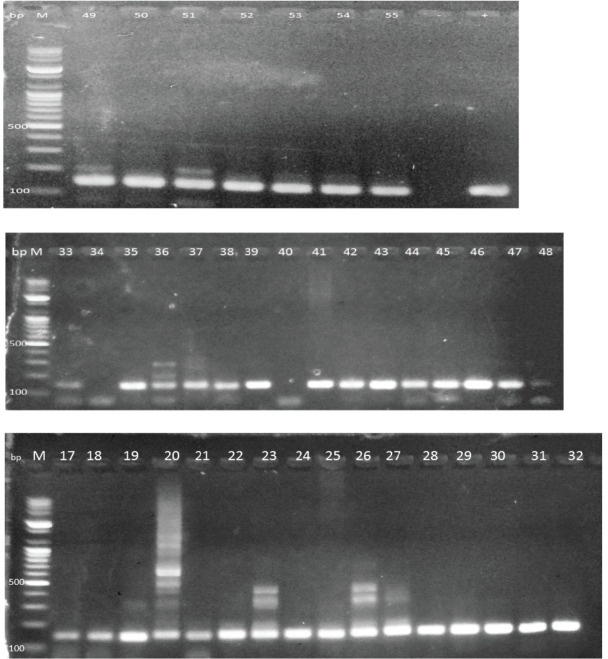
Electron micrographs showing HPV-DNA bands from DNA labs.
